# Simultaneous multi-slice excitation for single breath hold estimates of left ventricular rotational mechanics

**DOI:** 10.1186/1532-429X-18-S1-O109

**Published:** 2016-01-27

**Authors:** Zhe Wang, Fei Han, Yi Wang, Peng Hu, Daniel B Ennis

**Affiliations:** 1 Department of Bioengineering, University of California Los Angeles, Los Angeles, CA USA; 2 Department of Radiological Science, University of California Los Angeles, Los Angeles, CA USA; 3 Neurology, University of California, Los Angeles, CA USA

## Background

Quantitative measurements of left ventricular (LV) rotational mechanics can be estimated from cardiac tagged images and provide early insight into LV dysfunction. Current clinical protocols acquire tagged images at the LV apex and the LV base in two separate breath-held scans, which can contribute measurement bias due to differences in cardiac loading conditions. The first **objective** was to implement Simultaneous Multi-Slice (SMS) excitation (Felix A.B. *et al.* MRM 53:684-691 2005) for cardiac tagging to permit simultaneous acquisition of two short-axis grid tagged images. The second **objective** was to compare a conventional Multi Breath Hold (MBH) protocol to a Single Breath Hold (SBH) SMS protocol and evaluate the respective repeatability in healthy volunteers.

## Methods

A cardiac tagging sequence, which uses non-selective RF pulses to generate grid tag patterns throughout the heart, was modified to support SMS. The sequence was evaluated in an IRB approved study after obtaining consent from ten (N = 10) healthy human subjects with 15 heartbeat acquisition using the following parameters: 330-400 × 330-400 mm FOV, 6 mm slice thickness, TE/TR = 4.5/5.3 ms, 12° flip angle, 1.8 × 1.8 mm resolution, 250 Hz/pixel bandwidth, 8 phase encode lines per segment on a 1.5T scanner (Siemens Avanto). Simultaneous basal and apical images were acquired through mid-diastole (~750 ms). SMS images were reconstructed using a Matlab toolbox (Setsompop, K. *et al.* MRM 67, 1210-1224). Conventional MBH grid tags were also acquired. Each method was repeated twice. LV twist measurements were calculated using the FAST method (Reyhan M. *et al*. JMRI 2012 35:587-593). Both MBH and SBH acquisitions were repeated in random order. Repeatability results were compared using a t-test and Bland-Altman analysis.

## Results

**Figure 1:**
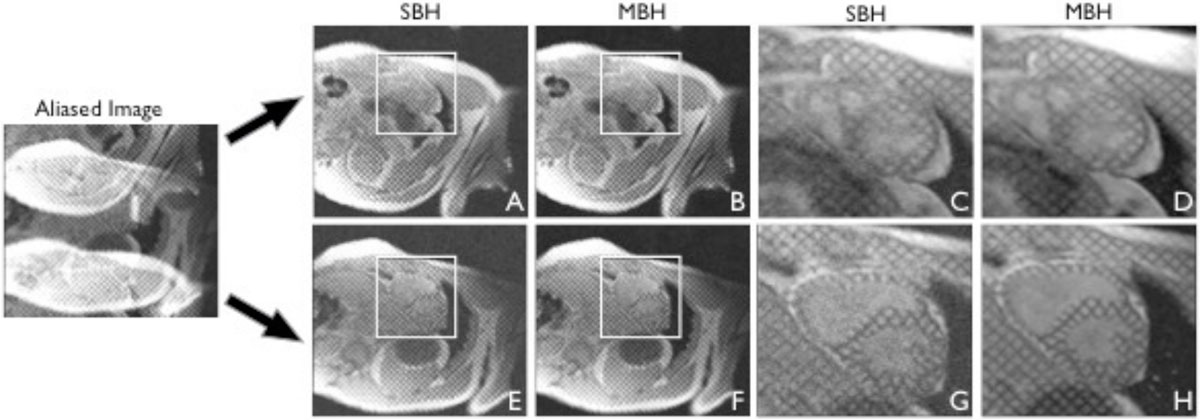
**Single breath hold (SBH)images before (aliased image) and after simultaneous multi-slice (SMS) reconstruction (A,E)**. Conventional multi-breath hold (MBH) images were used as the reference (B,F). A and B show the LV apical images while E and F show the LV basal images. C, D, G, and H correspond to the white box in A, B, E, and F respectively.

**Figure 2:**
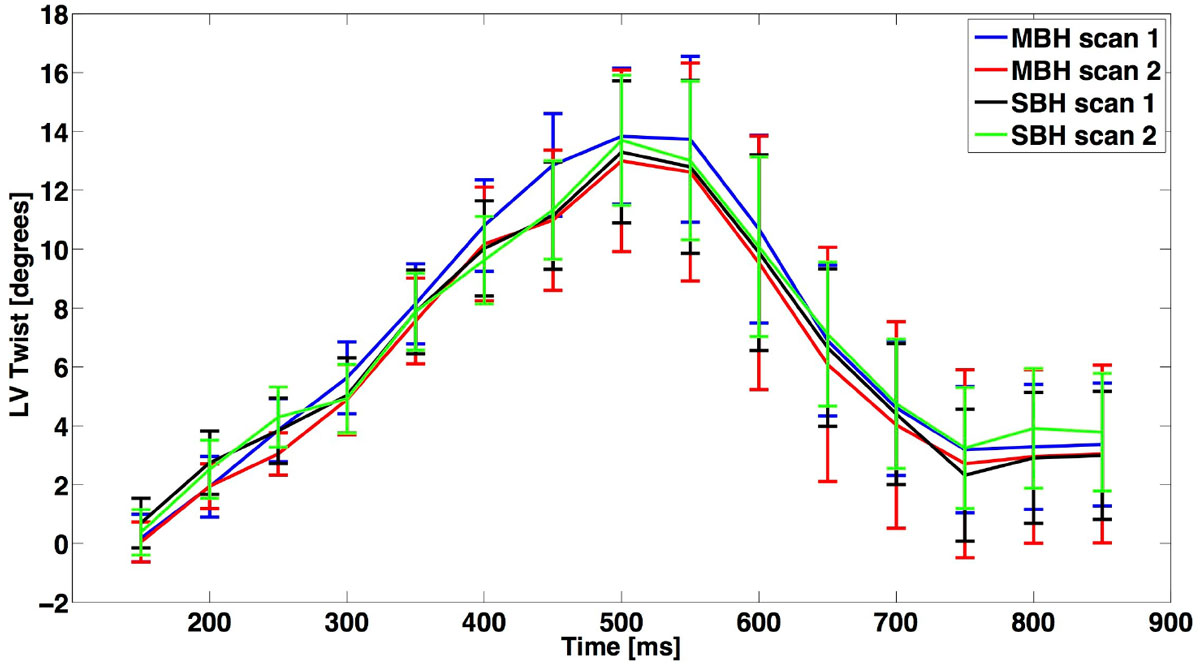
**LV twist during the cardiac cycle for conventional multi-breath hold (MBH) and single breath hold (SBH) with simultaneous multislice (SMS) excitation methods**. MBH and SBH LV twist curves are very similar, but the SBH method is acquired in half the scan time with improved repeatability.

Figure 1 shows the SBH LV tagged images before and after SMS reconstruction, and the conventional MBH grid tagged images. The reconstructed SBH SMS images showed good slice separation with slightly higher noise. Figure 2 shows the LV twist curve from SBH and MBH scans. The LV peak twist (LV-PT) results from the repeated measures for MBH and SBH were MBH #1 13.9° ± 2.3°, MBH #2 13.0° ± 2.9°, SBH #1 13.3° ± 2.4°, SBH #2 13.7° ± 2.2°. ΔLV-PT (repeatability) was higher for SBH compared to MBH and was significantly different (0.9° ± 0.6° vs. 0.4° ± 0.2°, p < 0.015). The Bland-Altman analysis of ΔLV-PT between SBH and MBH had a bias of 0.2° and 95% confidence intervals of [-0.5°, 0.9°]. The differences between the LV twist curves are small and the SBH curves are overlap more than MBH curves, which indicate a smaller bias.

## Conclusions

Since the tagging preparation pulses are non-selective, SMS and cardiac tagging can be combined into a single breath-held acquisition. The SBH SMS approach resulted in similar estimates of peak LV twist with improved repeatability in half the scan time compared to the MBH approach.

